# P58^IPK^ inhibition of endoplasmic reticulum stress in human retinal capillary endothelial cells in vitro

**Published:** 2008-07-13

**Authors:** Bin Li, Dong Li, Gui-gang Li, Hao-wen Wang, Ai-xia Yu

**Affiliations:** 1 Department of Ophthalmology, Tongji Hospital, Tongji Medical College, Huazhong University of Science and Technology, Wuhan, Hubei Province, People’s Republic of China; 2 State Key Laboratory of Ophthalmology, Zhongshan Ophthalmic Center, Sun Yat-sen University, Guangzhou, People’s Republic of China; 3 Network Research Division, Institute of Computing Technology, Chinese Academy of Sciences, Beijing, People’s Republic of China; 4 Central Hospital, E-Zhuo City, Hubei, People’s Republic of China

## Abstract

**Purpose:**

The goal of this research was to determine if P58^IPK^, a member of the Hsp40 family that inhibits eukaryotic initiation factor 2α (eIF2α), inhibits endoplasmic reticulum (ER) stress and leads to downregulated expression of vascular endothelial growth factor (VEGF) and decreased apoptosis in human retinal capillary endothelial cells (HRCECs).

**Methods:**

Recombinant vectors were constructed using P58 in adeno-associated virus type 2 (rAAV2-P58 ^IPK^) and P58 RNA in the plasmid pGIPZ (pGIPZ-P58^IPK^). The four experimental groups were: (1) non-transfected/non ER stressed control; (2) non-transfected/ER stressed; (3) rAAV2-P58^IPK^-transfected/ER stressed; and (4) pGIPZ- P58^IPK^ RNAi transfected/ER stressed. ER stress was induced by treating cells with tunicamycin. Expression of P58^IPK^ was determined in transfected cells. Expressions of the following factors were assessed: vascular endothelial growth factor (VEGF), C/EBP homologous protein (CHOP), activating transcription factor 4 (ATF4), and glucose-regulated protein 78 (GRP78). Apoptosis levels were also determined.

**Results:**

Significantly increased expression of P58^IPK^ was detected in cells transfected with rAAV2-P58^IPK^ (0.63±0.02) as compared to those transfected with pGIPZ-P58^IPK^ RNAi (0.23±0.01). P58^IPK^ expression was not different between the control transfected cells (rAAV2-GFP and pGIPZ-GFP). Following ER stress, expression levels of ATF-4, GRP78, CHOP, and VEGF in cells overexpressing P58^IPK^ were not different from those in unstressed control cells. This inhibitory effect of P58^IPK^ on the expression of ER stress-related factors was suppressed in cells transfected with pGIPZ-P58^IPK^ RNAi. Apoptosis was significantly increased in cells transfected with pGIPZ-P58^IPK^ RNAi but not with rAAV2-P58^IPK^.

**Conclusions:**

The study demonstrates that P58^IPK^ inhibits ER stress and plays an important role in maintaining balance and stability of the ER in HRCECs.

## Introduction

Endoplasmic reticulum (ER) stress is thought to be a contributing factor underlying many human diseases. In pathologic conditions, accumulation of unfolded or misfolded proteins in the ER can lead to ER stress or initiate the unfolded protein response [1,2]. If protein folding or degradation fails to occur in response, apoptotic pathways are initiated [1].

Studies in mice have indicated that ER stress can lead to islet cell apoptosis through induction of C/EBP homologous protein (CHOP) and contribute to the development of diabetes [3]. ER stress has also been implicated as a mediator of diabetic retinopathy (DR), a major microvascular complication of diabetes [4,5]. Roybal and colleagues [6] reported that ER stress increases the expression of vascular endothelial growth factor (VEGF) by activating the transcription factor 4 (ATF4) pathway. Further, Xu and colleagues [7] noted that both VEGF and protein kinase C expression were elevated in the early stages of DR using a rat model. These findings are significant as VEGF has been demonstrated to be involved in regulating a variety of pathologic changes associated with DR including neovascularization, vascular permeability, and thrombosis [8]. Given the evidence linking the development of DR with ER stress, it is pertinent to further elucidate the precise mechanisms/pathways involved so potential therapeutic interventions may be designed to treat/prevent DR by inhibiting ER stress.

P58^IPK^, a member of the Hsp40 family, was first discovered as an inhibitor of eukaryotic initiation factor 2α (eIF2α) [9]. Since this time, findings from several studies have indicated that P58^IPK^ plays an important role in ER stress protection [10,11]. For instance, Yan and colleagues [11] reported that decreases in P58^IPK^ activity were associated with increased expression of several ER stress-inducible genes such as *CHOP*. It has also been demonstrated that mice lacking the *P58^IPK^* gene exhibit gradual onset of glycosuria, hyperglycemia, and hypoinsulinemia [12]. The number of β-cells in the islets of these mice was significantly reduced, and the expressions of a variety of apoptosis-related genes were increased. This suggests that an apoptosis pathway is activated in the absence of P58^IPK^, leading to a decrease in the number of β-cells and loss of functionality. The ER stress-inhibiting effects of P58^IPK^ appear related to its capacity to inhibit phosphorylation of eIF2α, which is involved in eliciting the previously mentioned unfolded protein response [13].

To our knowledge, no study has examined the relationship between P58^IPK^ and ER stress in cells isolated from human eyes. Characterizing this relationship is important as it may help identify possible targets for pharmaceutical intervention (i.e., P58^IPK^ modulation) in the treatment/prevention of DR. In the present study, we sought to examine the effects of P58^IPK^ on ER stress in human retinal capillary endothelial cells (HRCECs) by assessing the expression of ER stress-related factors, glucose-regulated protein 78 (GRP78), ATF4, and CHOP. We also examined the possibility that P58^IPK^ may be able to ameliorate diabetes-associated blood vessel damage by assessing changes in VEGF expression and cellular apoptosis. Our working hypothesis was that P58^IPK^ would inhibit ER stress in HRCECs.

## Methods

### Human retinal capillary endothelial cells culture and characterization

Donor eyes (12) were obtained from the Tongji Hospital Eye Bank. Donors were healthy accident victims with an average age of 33.5 years (consent was granted by the donors’ family members and experiments were approved by the Internal Review Board of the hospital).

The well established method for isolating and culturing HRCECs has been previously described [14]. The donor eyes were cut circumferentially 3 mm posterior to the limbus. Retinas were then harvested and homogenized by two gentle up/down strokes in a 15 ml Dounce homogenizer (Chuang Rui company, Dongguan, China). Collected cells were resuspended in human endothelial serum-free material (SFM) basal growth medium (Gibco Grand Island, NY), supplemented with 20% fetal bovine serum (FBS), 50 U/ml endothelial cell growth factor (Sigma-Aldrich, St. Louis, MO), and 1% insulin–transferrin–selenium (Gibco, Inc. Los Angeles, CA). Cells were cultured and passaged in fibronectin-coated dishes at 37 °C in a humidified atmosphere containing 5% CO_2_. Cultured cells were then characterized for endothelial homogeneity by testing for activity to factor VIII antigen (von Willebrand factor), measuring acetyl-LDL (Ac-LDL) receptors in endothelial cells, as described previously [14], and by morphological examination by light microscopy. Only cells from passages 3-7 were used in the experiments.

HRCECs were treated with tunicamycin (an ER stressor) according to a modification of the method described by Shimazawa and colleagues [15]. Cells were washed twice with Dulbecco’s Modified Eagle Medium (DMEM; Sigma-Aldrich) and then immersed in DMEM supplemented with 1% FBS plus tunicamycin (2 μg/ml) for 12 h. Cell viability was measured using a single-cell digital imaging-based method that stained nuclei. Retinal fluorescence intensity was visualized in vivo.

### Construction and packaging of recombinant adeno-associated virus type 2- (rAAV2) P58^IPK^

The coding sequence of p58^IPK^ was obtained from the human cDNA bank/domain and synthesized using Golden Taq (Tiangen Biotech Beijing, China). pSNAV- P58^IPK^ plasmids were then formed after DNA digestion and ligation. Transfection of BHK-21 cells with pSNAV-P58^IPK^ was performed in a 6 well plate using a Lipofectamine 2000 kit (Invitrogen Inc, Carlsbad, CA). These cells are hereafter referred to as BHK/P58^IPK^. BHK/P58^IPK^ cells were then transferred to and cultured in a flask (110x480 mm; Wheaton Inc, Millville, NJ) before being infected with HSV1-rc/ΔUL2 (Benyuan Zhengyang Inc, Beijing, China; moore=0.1) to induce rAAV2-P58^IPK^ when the cell number reached 8×10^8^. The cells were detached after 48 h of culture and were then divided into Fernbach culture flasks (250 ml) for further purification. Purified rAAV2-P58^IPK^ was identified by reverse-transcription polymerase chain reaction (RT–PCR). The titer of rAAV2-P58^IPK^ (virus genome/ml) was assessed using in situ hybridization with a digoxin labeled cucumber mosaic virus probe to obtain a final titer of 1×10^12^.

### Construction of pGIPZ-P58 ^IPK^ RNAi using pGIPZ, a lentiviral shRNA cloning vector

The P58^IPK^ RNAi (Addgene, Inc., Cambridge, MA) sequence inserted was as follows: TGC TGT TGA CAG TGA GCG CAG GTG CTG AAT GTG GAG TAA ATA GTG AAG CCA CAG ATG TAT TTA CTC CAC ATT CAG CAC CTT TGC CTA CTG CCT CGG A. pGIPZ-green fluorescence protein (GFP) was used to evaluate transfection efficiency.

### Plasmid transfection of human retinal capillary endothelial cells

Cultured HRCECs were transfected with rAAV2-P58^IPK^, PGIPZ-P58^IPK^ RNAi, pGIPZ-GFP, or rAAV2-GFP. Cells were plated in 24 well culture plates, grown overnight to 70%-80% confluence, and then washed twice with serum-free basal growth medium. While only rAAV2 was added to control group wells, 10×10^9^ copies of rAAV2- P58^IPK^, or PGIPZ- P58^IPK^ RNAi were added to each well in the experimental groups. In addition, plasmids containing rAAV2-GFP and pGIPZ-GFP were separately added to HRCECs to monitor transfection efficiency.

### Semi-quantitative PCR and real time PCR for P58^IPK^ expression in human retinal capillary endothelial cells

HRCECs were harvested, and RNA was extracted using the QiagenRNA extraction kit (Qiagen Inc., Venlo, The Netherlands). After quantification, RNA was reverse transcribed to cDNA using the Super Script II RT kit (Invitrogen Inc.). Real time PCR was performed using the Hotstar Taq Polymerase kit (Qiagen Inc.) with SYBR Green technology (ABI Inc., Foster City, CA). GAPDH was used as an internal control. Primers for GAPDH were as follows: sense, 5′-CCT GTA CGC CAA CAC AGT GC-3′ and antisense, 5′-ATA CTC CTG CTT GCT GAT CC-3′. Primers for P58^IPK^ were as follows: sense, 5′-GAG GTT TGT GTT GGG ATG CAG-3′ and antisense, 5′-GCT CTT CAG CTG ACT CAA TCA G-3′. The PCR reaction for P58^IPK^ was performed in a final reaction volume of 20 μl using the following conditions: a preheating cycle at 95 °C for 3 min then 31 cycles at 95 °C for 30 s, 62 °C for 30 s, and 72 °C for 30 s followed by an elongation cycle at 72 °C for 8 min. Melting curve analysis was performed by monitoring FAM/SYBR fluorescence.

For semi quantitative (sq) PCR, 1 μl of single-stranded cDNA was added to a 20 μl PCR reaction mixture containing 10 pmol of a gene specific primer pair, 1X PCR buffer (Invitrogen Inc.) containing 3μM MgCl_2_, 5μM dNTPs, and 1 unit of Platinum Taq (Invitrogen Inc.). PCR conditions were as follows: initial denaturation at 95 °C for 4 min, 30 cycles at 95°C for 30 s, 58°C for 45 s, and 72°C for 30 s, the final elongation step was performed for 5 min at 72°C. After 30 cycles, the final elongation step was performed for 5 min. Equal amounts of PCR products were separated on 1.5% agarose gels and visualized by ethidium bromide staining. GAPDH was used as an internal control.

### Cell apoptosis analysis

There were a total of four experimental groups in this study: (1) a control group (no ER stress or transfection); (2) an ER stress group (no transfection); (3) a P58^IPK^ transfection (upregulation)/ER stress group; and (4) a P58^IPK^ RNAi (inhibition)/ER stress group. cellular apoptosis was evaluated using propidium iodide (PI) to stain the DNA. Cells were trypsinized, pelleted, and resuspended in one volume of ice-cold phosphate-buffered saline (PBS). The suspension was gently agitated while three volumes of ice-cold 95% ethanol were slowly added. Cells were then pelleted and resuspended in equal volumes of 30 μg/ml PI and 100 μg/ml RNase A (both in PBS). Stained cells were stored overnight at 4 °C and protected from light until analysis. Flow cytometry was performed to determine the percentage of cells in apoptosis.

### Real time PCR for gene expression of VEGF, GRP78, ATF4, and CHOP

HRCECs from each experimental group were collected for detection of VEGF, GRP78, ATF4 and CHOP expression. Total RNA was isolated for quantification. RNA was then reverse transcribed to cDNA using a Super Script II RT kit (Invitrogen Inc). Real time PCR was performed using the Hotstar Taq Polymerase kit (Qiagen Inc.) with SYBR Green technology (ABI Inc.). Primers for VEGF, GRP78, ATF4, CHOP, and GAPDH are described in Table 1. The PCR reaction was performed in a final reaction volume of 20 μl under the following conditions: a preheating cycle at 95 °C for 5 min then 33 cycles at 95°C for 20 s, 56°C for 30 s, and 72°C for 30 s, the final elongation step was performed for 5 min at 72°C an elongation cycle at 72 °C for 8 min. Melting curve analysis was performed by monitoring FAM/SYBR fluorescence.

**Table 1:** Human retinal capillary endothelial cells real time PCR primers

**Gene**	**Primer (5’-3’)**	**PCR annealing temp (°C)**
*VEGF*	GACAAGAAAATCCCTGTGGGC	56
	AACGCGAGTCTGTGTTTTTGC	
*GRP78*	GCCTGTATTTCTAGACCTGCC	56
	TTCATCTTGCCAGCCAGTTG	
*CHOP*	AGAGGAAGAATCAAAAACCTTCACT	56
	ACTCTGTTTCCGTTTCCTAGTTCTT	
*ATF4*	AGGAGTTCGCCTTGGATGCCCTG	56
	AGTGATATCCACTTCACTGCCCAG	
*GAPDH*	CCTGTACGCCAACACAGTGC	56
	ATACTCCTGCTTGCTGATCC	

### Western blotting

Total protein was extracted from the HRCECs and quantified using bicinchoninic acid. The protein was mixed with the loading buffer, denatured for 6 min at 60 °C, cooled, centrifuged for 5 min, and then separated by sodium-dodecyl sulfate PAGE (SDS–PAGE). Monoclonal anti-CHOP (Santa Cruz Biotechnology Inc., Santa Cruz, CA), polyclonal anti-ATF4 (Santa Cruz Biotechnology, Inc.), monoclonal anti-GRP78 (BD Biosciences, San Jose, CA),and monoclonal anti-VEGF (Santa Cruz Biotechnology Inc.) were used to probe the proteins. A secondary antibody (1:1000 dilution) was then applied, and the signal was revealed by chemiluminescence. The same polyvinylidene fluoride (PVDF) membranes were reused to detect β-actin (internal control) by incubating with the mouse anti-human actin antibody (Gene Co., Hong Kong, China). The bands observed on the films were analyzed by automatic image analysis, and the integrated optical density (OPTDI) of each protein band was normalized to the OPTDI value of the corresponding β-actin band from the same sample.

### Statistical analysis

Normally distributed data were compared by one-way ANOVA while non-normally distributed data were compared by Kruskal–Wallis test. When significant differences were detected between groups, multiple comparisons of means were performed using the Bonferroni procedure, with type-I error rate at a maximum of 0.008 (0.05/6) adjustment. Statistical analyses were performed using SPSS 15.0 Statistics software (SPSS Inc., Chicago, IL), and differences were considered significant when p<0.05. Data are presented as the mean±standard error of the mean (SEM).

## Results

**Figure 1:**
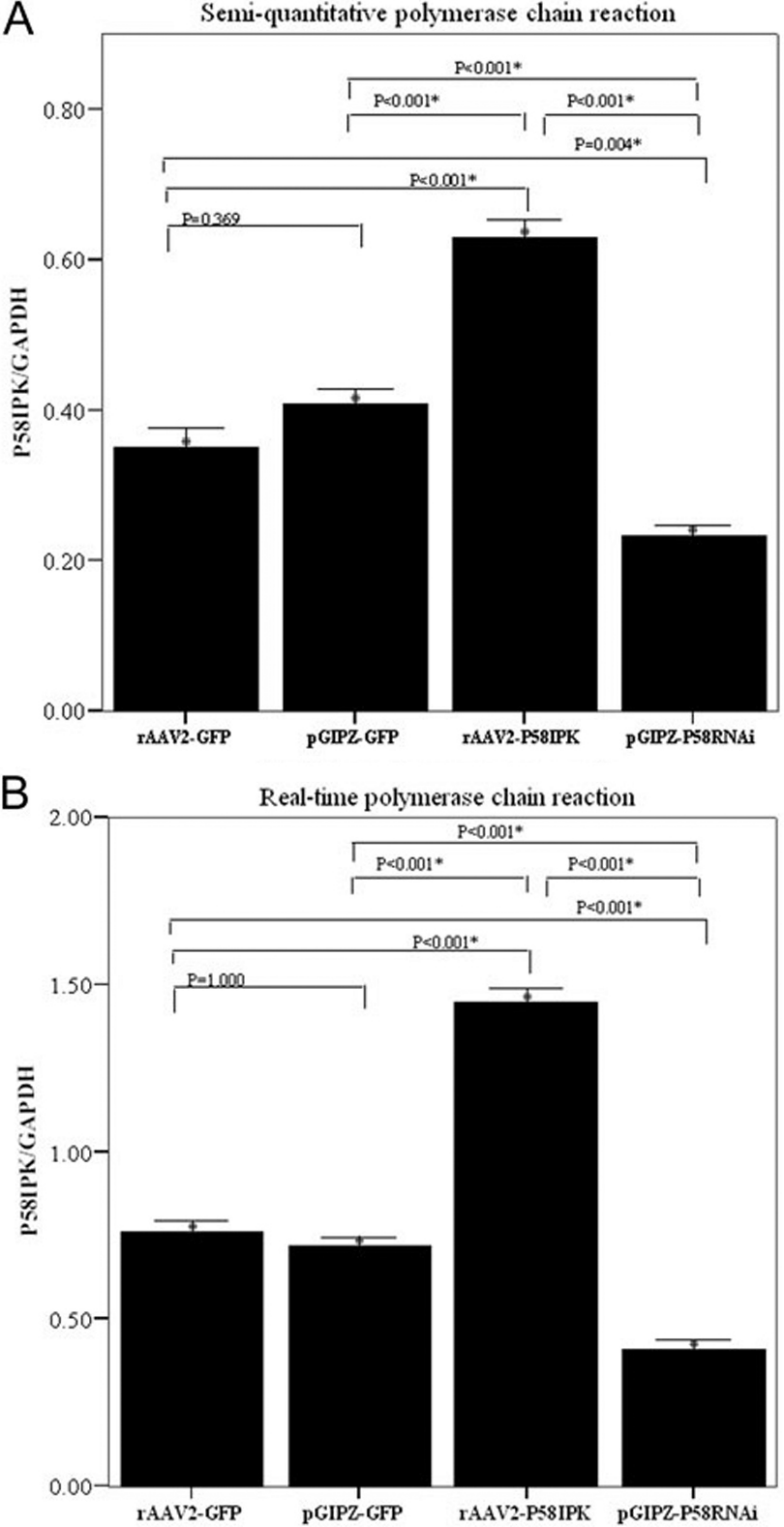
P58^IPK^ expression in human retinal capillary endothelial cells in different groups**. A:** P58IPK expression in human retinal capillary endothelial cells (HRCECs) transfected with rAAV2-GFP, pGIPZ-GFP, rAAV2- P58^IPK^, and pGIPZ-P58^IPK^ RNAi as determined by semi-quantitative PCR. There were significant differences in the expression levels of P58^IPK^/GAPDH among the four transfection groups (p<0.001). The highest level was in the group transfected with rAAV2- P58^IPK^ (0.63±0.04) while the lowest was in the group with pGIPZ- P58^IPK^ RNAi (0.23±0.04). No significant differences in P58^IPK^/GAPDH levels were found between the rAAV2-GFP (0.35±0.05) and pGIPZ-GFP groups (0.41±0.04). **B:** P58^IPK^ expression in (HRCECs) transfected with rAAV2-GFP, pGIPZ-GFP, rAAV2- P58^IPK^, and pGIPZ-P58^IPK^ RNAi as determined by real time PCR. There were significant differences in the expression levels of P58^IPK^/GAPDH among the four transfection groups (p<0.001). The ratio of P58^IPK^/GAPDH was 1.45±0.09 in the rAAV2- P58^IPK^ group and 0.41±0.06 in the pGIPZ- P58^IPK^ RNAi group. No significant differences in P58^IPK^/GAPDH levels were found between the rAAV2-GFP (0.76±0.05) and pGIPZ-GFP groups (0.72±0.05).

The transfection rate of rAAV2-P58IPK was 85% and pGIPZ- P58IPK RNAi was 87% two days after transfection (transfection rates of the P58IPK and GFP plasmids were assumed to be equivalent). Gene expression levels of P58^IPK^ in HRCECs transfected with rAAV2-GFP, pGIPZ-GFP, rAAV2- P58^IPK^, or pGIPZ- P58^IPK^ RNAi as determined by semi-quantitative polymerase chain reaction (sq PCR) and real time PCR are presented in Figure 1. There were significant differences in the expression levels of P58^IPK^/GAPDH among the four transfection groups (p<0.001). sq PCR result show that the highest level was in the group transfected with rAAV2- P58^IPK^ (0.63±0.02) while the lowest was in the group with pGIPZ- P58^IPK^ RNAi (0.23±0.01). No significant differences in P58^IPK^/GAPDH levels were found between the rAAV2-GFP and pGIPZ-GFP groups. The results of real time PCR show that the ratio of P58IPK/GAPDH was 1.45±0.05 in the rAAV2- P58^IPK^ group and 0.41±0.03 in the pGIPZ- P58^IPK^ RNAi group.

**Figure 2:**
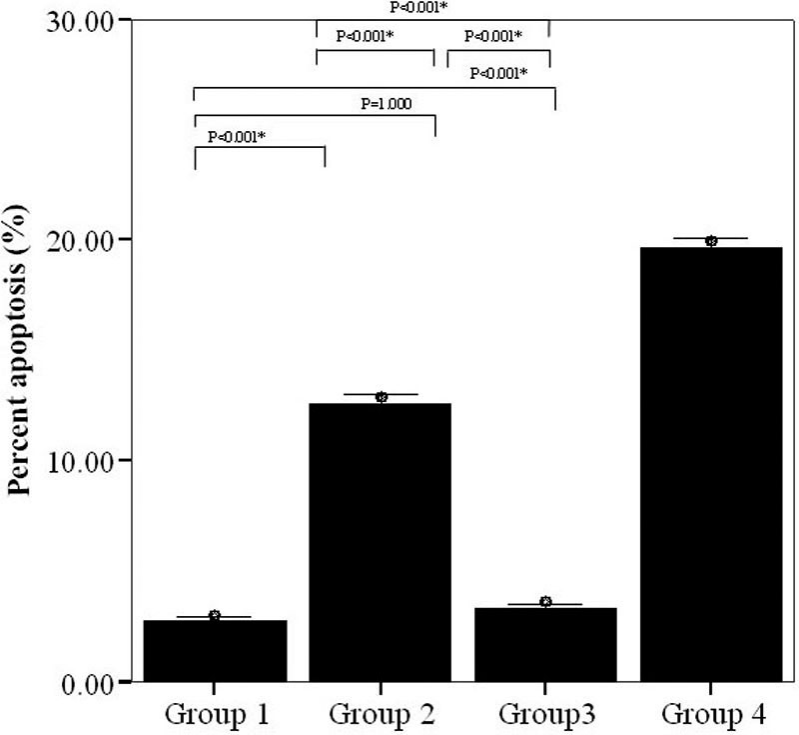
Effect of P58^IPK^ on apoptosis in human retinal capillary endothelial cells s after tunicamycin-induced endoplasmic reticulum stress. There was no significant difference between groups 1 (control) and 3 (rAAV2- P58^IPK^). Apoptosis levels were higher than groups 1 and 3 in group2. Apoptosis levels were significantly higher in group 4 (co-transfected with pGIPZ- P58^IPK^ RNAi and rAAV2- P58^IPK^) compared to all other groups (p<0.001 for all). Group 1 represents normal control that was not endoplasmic reticulum-stressed. Group 2 represents human retinal capillary endothelial cells (HRCECs) treated with tunicamycin. Group 3 represents HRCECs transfected with rAAV2-P58^IPK^ and treated with tunicamycin. Group 4 represents HRCECs transfected with pGIPZ-P58^IPK^ RNAi and treated with tunicamycin.

There was a statistically significant overall difference in apoptosis levels two days after transfection (Figure 2). There was no significant difference between groups 1 (control) and 3 (rAAV2- P58^IPK^). Apoptosis levels were significantly higher in group 4 (co-transfected with pGIPZ- P58^IPK^ RNAi and rAAV2- P58^IPK^) compared to all other groups (p<0.001 for all).

**Figure 3:**
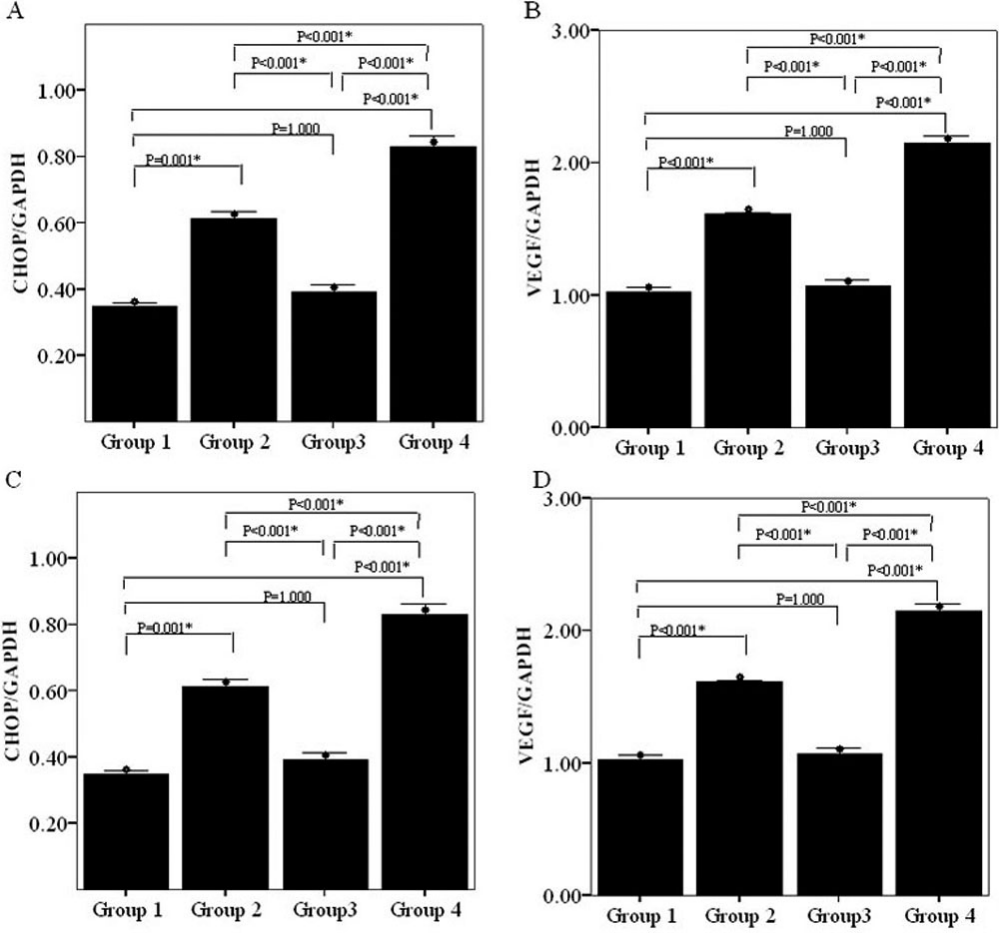
The effect of P58^IPK^ on gene expression of ATF4, C/EBP homologous protein, GRP78, and vascular endothelial growth factor on human retinal capillary endothelial cells by real time PCR. **A:** Evaluation the effect of P58^IPK^ on gene expression of ATF4 on human retinal capillary endothelial cells (HRCECs) treated with tunicamycin by real time PCR. Group 1 is normal control that was not endoplasmic reticulum (ER)-stressed. Group 2 is HRCECs treated with tunicamycin. Group 3 is HRCECs transfected with rAAV2-P58^IPK^, and treated with tunicamycin. Group 4 is HRCECs transfected with pGIPZ-P58^IPK^ RNAi and treated with tunicamycin. The expression level of ATF4 in either group was: 0.52±0.05, 1.14±0.05, 0.58±0.06, and 1.54±0.06 (p< 0.001), respectively. **B:** Evaluation the effect of P58^IPK^ on gene expression of GRP78 on HRCECs treated with tunicamycin by real time PCR: Group 1 represents normal control that was not ER-stressed. Group 2 represents HRCECs treated with tunicamycin. Group 3 represents HRCECs transfected with rAAV2-P58^IPK^ and treated with tunicamycin. Group 4 represents HRCECs transfected with pGIPZ-P58^IPK^ RNAi and treated with tunicamycin. The expression level of GRP78 in either group was: 1.13±0.08, 1.96±0.14, 1.09 ±0.13, and 2.44±0.16 (p< 0.001), respectively. **C:** Evaluation the effect of P58^IPK^ on gene expression of C/EBP homologous protein (CHOP) on HRCECs treated with tunicamycin by real time PCR. Group 1 is normal control that was not ER-stressed. Group 2 is HRCECs treated with tunicamycin. Group 3 is HRCECs transfected with rAAV2-P58^IPK^ and treated with tunicamycin. Group 4 is HRCECs transfected with pGIPZ-P58^IPK^ RNAi and treated with tunicamycin. The expression level of CHOP in either group was: 0.35±0.02, 0.61±0.03, 0.39±0.04, and 0.83±0.06 (p< 0.001), respectively. **D:** Evaluation the effect of P58^IPK^ on gene expression of vascular endothelial growth factor (VEGF) on HRCECs treated with tunicamycin by real time PCR. Group 1 represents normal control that was not ER-stressed. Group 2 represents HRCECs treated with tunicamycin. Group 3 represents HRCECs transfected with rAAV2-P58^IPK^ and treated with tunicamycin. Group 4 represents HRCECs transfected with pGIPZ-P58^IPK^ RNAi and treated with tunicamycin. The expression level of VEGF in either group was: 1.02±0.07, 1.61±0.04, 1.02 ±0.07, and 2.14±0.12 (p< 0.001), respectively.

**Figure 4:**
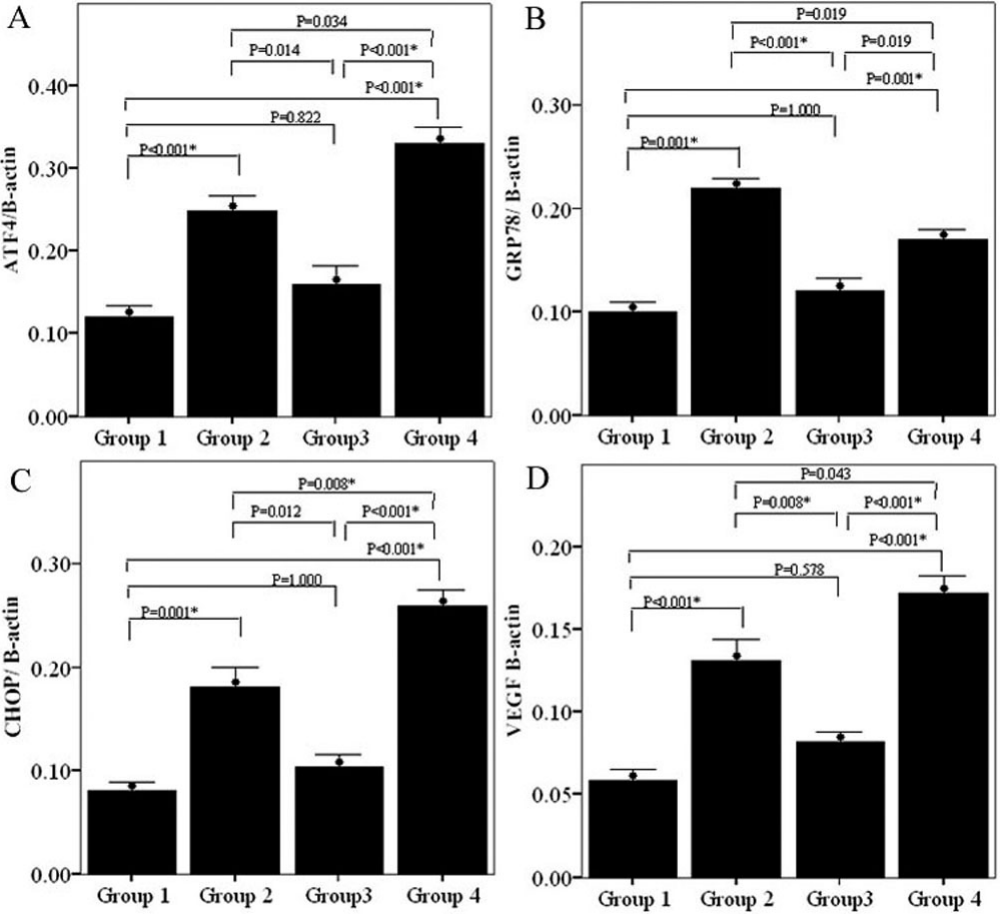
The effect of P58^IPK^ on gene expression of ATF4, C/EBP homologous protein, GRP78 and vascular endothelial growth factor on human retinal capillary endothelial cells by western blot. **A:** Evaluation the effect of P58^IPK^ on gene expression of ATF4 on human retinal capillary endothelial cells (HRCECs) treated with tunicamycin by western blot: Group 1 is normal control that was not ER-stressed. Group 2 is HRCECs treated with tunicamycin. Group 3 is HRCECs transfected with rAAV2-P58^IPK^ and treated with tunicamycin. Group 4 is HRCECs transfected with pGIPZ-P58^IPK^ RNAi and treated with tunicamycin. The expression level of ATF4 in either group was: 0.12±0.03, 0.25±0.03, 0.16±0.04, and 0.33±0.04 (p< 0.001), respectively. **B:** Evaluation the effect of P58^IPK^ on gene expression of GRP78 on HRCECs treated with tunicamycin by western blot: Group 1:normal control that was not endothelial reticulum (ER)-stressed. Group 2 is HRCECs treated with tunicamycin. Group 3 is HRCECs transfected with rAAV2-P58^IPK^ and treated with tunicamycin. Group 4 is HRCECs transfected with pGIPZ-P58^IPK^ RNAi and treated with tunicamycin. The expression level of GRP78 in either group was: 0.10±0.02, 0.22±0.02, 0.12±0.03, and 0.17±0.02 (p< 0.001), respectively. **C:** Evaluation the effect of P58^IPK^ on gene expression of C/EBP homologous protein (CHOP) on HRCECs treated with tunicamycin by western blot: Group 1 is normal control that was not ER-stressed. Group 2 is HRCECs treated with tunicamycin. Group 3 is HRCECs transfected with rAAV2-P58^IPK^ and treated with tunicamycin. Group 4 is HRCECs transfected with pGIPZ-P58^IPK^ RNAi and treated with tunicamycin. The expression level of CHOP in either group was: 0.08±0.02, 0.18± 0.04, 0.10 ±0.03, and 0.26±0.03 (p< 0.001), respectively. **D:** Evaluation the effect of P58^IPK^ on gene expression of vascular endothelial growth factor (VEGF) on HRCECs treated with tunicamycin by western blot: Group 1 is normal control that was not ER-stressed. Group 2 is HRCECs treated with tunicamycin. Group 3 is HRCECs transfected with rAAV2-P58^IPK^ and treated with tunicamycin. Group 4 is HRCECs transfected with pGIPZ-P58^IPK^ RNAi and treated with tunicamycin. The expression level of VEGF in either group was: 0.06±0.01, 0.13±0.02, 0.08±0.01, and 0.17±0.02 (p< 0.001), respectively.

**Figure 5:**
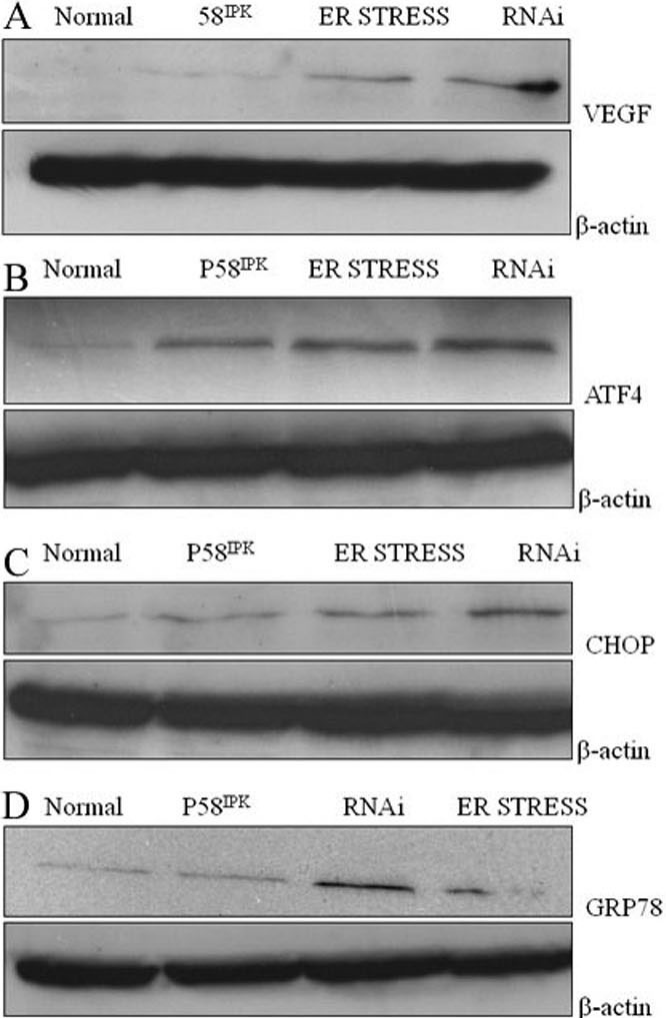
The effect of P58^IPK^ on gene expression of ATF4, C/EBP homologous protein, GRP78 and vascular endothelial growth factor on human retinal capillary endothelial cells by western blot. **A:** Evaluation the effect of P58IPK on gene expression of vascular endothelial growth factor (VEGF) on human retinal capillary endothelial cells (HRCECs) treated with tunicamycin by western blot. Normal represents normal control that was not endothelial reticulum (ER)-stressed; P58^IPK^ represents HRCECs transfected with rAAV2-P58^IPK^ and treated with tunicamycin ; ER stress represents HRCECs treated with tunicamycin; RNAi represents HRCECs transfected with pGIPZ-P58^IPK^ RNAi, and treated with tunicamycin. The expression level of VEGF in either group was: 0.06±0.01, 0.08±0.01, 0.13±0.02; and 0.17±0.02 (p<0.001), respectively. **B:** Evaluation the effect of P58^IPK^ on gene expression of ATF4 on HRCECs treated with tunicamycin by western blot: Normal represents normal control that was not ER-stressed; P58^IPK^ represents HRCECs transfected with rAAV2-P58^IPK^ and treated with tunicamycin ;ER stress represents HRCECs treated with tunicamycin;RNAi represents HRCECs transfected with pGIPZ-P58^IPK^ RNAi and treated with tunicamycin. The expression level of ATF4 in either group was: 0.12±0.03, 0.16±0.04, 0.25±0.03, and 0.33±0.04 (p<0.001), respectively. **C:** Evaluation the effect of P58^IPK^ on gene expression of C/EBP homologous protein (CHOP) on HRCECs treated with tunicamycin by western blot. Normal represents normal control that was not ER-stressed; P58^IPK^ represents HRCECs transfected with rAAV2-P58^IPK^ and treated with tunicamycin; ER stress represents HRCECs treated with tunicamycin; RNAi represents HRCECs transfected with pGIPZ-P58^IPK^ RNAi and treated with tunicamycin. The expression level of CHOP in either group was: 0.08±0.02, 0.10±0.03, 0.18±0.04, and 0.26±0.03 (p< 0.001), respectively. **D:** Evaluation the effect of P58^IPK^ on gene expression of GRP78 on HRCECs treated with tunicamycin by western blot. Normal represents normal control that was not ER-stressed; P58^IPK^ represents HRCECs transfected with rAAV2-P58^IPK^ and treated with tunicamycin; RNAi represents HRCECs transfected with pGIPZ-P58^IPK^ RNAi and treated with tunicamycin; ER stress represents HRCECs treated with tunicamycin. The expression level of GRP78 in either group was: 0.10±0.02, 0.12±0.03, 0.22±0.02, and 0.17±0.02 (p<0.001), respectively.

Significant differences in both gene and protein expression of ATF4, GRP78, CHOP, and VEGF were apparent between the four groups as determined by real time PCR and western blot analyses, respectively (p<0.001, see Figure 3 and Figure 4). Real time PCR revealed that expression levels of ATF4, GRP78, CHOP, and VEGF were significantly increased in the group treated with tunicamycin (Group 2) compared to the normal control group (Group 1, p<0.001 for all comparisons). Expression of all four genes were highest in Group 4 cells (those co-transfected with rAAV2- P58^IPK^ and pGIPZ- P58^IPK^ RNAi and treated with tunicamycin). In contrast, expression levels of these genes in Group 3 cells (transfected with rAAV2- P58^IPK^ and treated with tunicamycin) were not significantly different from those in control cells (Group 1, Figure 3). Protein levels of ATF4, GRP78, CHOP, and VEGF were significantly increased in the groups treated with tunicamycin (Group 2) as well as groups that co-transfected with rAAV2- P58^IPK^ and pGIPZ- P58^IPK^ RNAi and treated with tunicamycin (Group 4) when compared to the normal control group (Group 1, Figure 4). The protein expression levels of ATF4, CHOP, and VEGF in Group 4 cells (co-transfected with rAAV2- P58^IPK^ and pGIPZ- P58^IPK^ RNAi) were also significantly higher than those in Group 3 (transfected with rAAV2- P58^IPK^). No significant differences were detected between Group 3 (transfected with rAAV2- P58^IPK^ and treated with tunicamycin) and Group 1 (normal control group) for these variables (Figure 4). Representative western blot images are shown in Figure 5.

## Discussion

ER stress is known to cause apoptosis in retinal cells. In the early stages of DR, increased expression of CHOP, GRP78, and other factors closely correlate with apoptosis in retinal pericytes [5]. Hence, it has been suggested that ER stress plays a key role in the initiation and development of DR.

Taking into account previously reported findings; we conducted a study of HRCECs transfected with P58^IPK^ to examine the effects of P58^IPK^ on the relief of ER stress (a mediator of diabetes and DR) and apoptosis. We found that HRCECs overexpressing P58^IPK^ exhibited decreased expression of the ER stress-related factors, GRP78, ATF4, and CHOP, following ER stress. Importantly, when P58^IPK^ expression was silenced by co-transfecting HRCECs with RNAi against P58^IPK^, the previously noted inhibition of ER stress was abolished i.e., expression of the aforementioned factors were increased. These findings suggest that P58^IPK^ plays an important role in maintaining the balance and stability of the ER in HRCECs.

P58^IPK^ is purported to be an important factor in maintaining balance within the endoplasmic reticulum [16]. In the present study, we demonstrated that when endogenous P58^IPK^ expression was increased by transfection, expression of ATF4 and VEGF was decreased in HRCECs following ER stress. Given these findings, we speculate that P58^IPK^ has the capacity to downregulate the activity of the ATF4/VEGF pathway during ER stress. We also noted that increased P58^IPK^ expression was associated with decreased CHOP expression in HRCECs. Given that CHOP is an initiator of apoptosis, it is hardly surprising that apoptosis levels also decreased in cells that overexpressed P58^IPK^. With regards to the development of DR, the current findings lead us to suggest that enhanced P58^IPK^ expression may help to decelerate or prevent the process of retinal vessel damage caused by upregulated VEGF expression.

In summary, to our knowledge, this is the first paper to describe the effects of P58^IPK^ on ER stress in human retinal capillary endothelial cells in vitro. The results of the present study indicate that the overexpression of P58^IPK^ in HRCECs can effectively ameliorate ER stress by inhibiting expressions of GRP78, ATF4, VEGF, and CHOP and decreasing apoptosis. Further study is warranted to clarify precisely how P58^IPK^ interacts with VEGF-biologic pathways during ER stress. Confirmation of these findings using an in vivo animal model is also needed.
